# Immune markers characteristic for asymptomatically infected and diseased *Entamoeba histolytica* individuals and their relation to sex

**DOI:** 10.1186/s12879-014-0621-1

**Published:** 2014-11-25

**Authors:** Hannah Bernin, Claudia Marggraff, Thomas Jacobs, Norbert Brattig, Le Van An, Jörg Blessmann, Hannelore Lotter

**Affiliations:** Bernhard Nocht Institute for Tropical Medicine, Bernhard-Nocht-Str. 74, Hamburg, 20359 Germany; Medical College, Hue' University of Medicine, 6 Ngo Quyen St., Hue City, Vietnam; Department of Molecular Parasitology, Bernhard Nocht Institute for Tropical Medicine, Bernhard-Nocht-Str. 74, Hamburg, 20359 Germany

**Keywords:** Entamoeba histolytica, Asymptomatic carrier, Amebic liver abscess, Immune response, Sex difference

## Abstract

**Background:**

The protozoan parasite *Entamoeba histolytica* (*E. histolytica*) usually asymptomatically colonizes the human intestine. In the minority of the cases, the parasite evades from the gut and can induce severe symptoms like colitis or amebic liver abscess (ALA). Interestingly, ALA predominates in adult men despite a higher prevalence of the parasite in women. The present study aimed to identify characteristic serum markers in a unique cohort of clearly defined asymptomatically infected *E. histolytica* individuals in comparison to patients with an *E. histolytica* liver manifestation of both sex.

**Methods:**

The following study groups were investigated: ALA patients (n = 38), healthy asymptomatic *E. histolytica* carriers (AC) (n = 44), and healthy *E. dispar*-infected controls (n = 24) out of an amebiasis endemic area. *E. histolytica*-specific immunoglobulin G (IgG) and the IgG subclasses against proteinaceous and non-proteinaceous amebic antigens were measured by ELISA. Serum cytokine and chemokine levels were investigated using a flow cytometry bead-based multiplex immunoassay.

**Results:**

The IgG results revealed that not only ALA patients, but also AC, developed high *E. histolytica-*specific titers of IgG and all IgG subclasses as well as IgA. IgG and IgG2 titers against the glycolipid *E. histolytica* lipophosphoglycan were highest in ALA patients. As in ALA patients, high cytokine levels of interleukin (IL-) 4 were detected in AC compared to *E. dispar* infected individuals, while IL-6 was exclusively elevated in ALA patients. IL-10 was lower in AC compared to ALA patients. Equal serum levels of CCL2 were found in all study groups but ALA patients showed decreased levels of CCL3. Sex dependent analysis of the data indicated significantly higher IgG and IgG1 titers in female AC compared to male AC. CCL2, the chemokine involved in immunopathology in the mouse model for the disease, was higher in male AC compared to female AC.

**Conclusion:**

In this study we characterize for the first time an asymptomatic carrier stage in amebiasis that is associated with a significant immune reaction and provide immunological markers that might give first hints towards an understanding of immune mechanisms underlying the control or development of invasive amebiasis.

**Electronic supplementary material:**

The online version of this article (doi:10.1186/s12879-014-0621-1) contains supplementary material, which is available to authorized users.

## Background

The protozoan parasite *Entamoeba histolytica* (*E. histolytica)* is the causative agent of invasive amebiasis, a disease that represents a major health problem in subtropical and tropical areas as well as in returnees from amebiasis-endemic areas [1],[2]. The parasite colonizes the bowel system of its host for months or years without inducing clinical symptoms of disease [3]. Only in about 10% of the cases, the parasite evades from the gut leading to severe clinical disorders like hemorrhagic colitis or, in case of a spread via the blood stream, a destruction of the liver tissue, the amebic liver abscess (ALA). In contrast to amebic colitis and despite similar or even higher infection rates in women, ALA mainly occurs in adult men [4]-[6]. The host-dependent immune mechanisms that are either involved in the ability to restrict an infection to the intestine or that might be responsible for the development of severe disease are not known.

Individuals that are asymptomatically infected with *E. histolytica* could represent an important group enabling the study of immune responses that are critical to the outcome of an infection. However, the identification of such individuals requires a diagnostic tool that can distinguish *E. histolytica* gut infections from infections with the morphologically identical but harmless intestinal parasite *Entamoeba dispar* (*E. dispar)*, which is widely distributed in regions endemic for *E. histolytica* [7]. *E. dispar* has been responsible for screening errors in the past. To address this, a highly sensitive and specific real-time PCR was developed that allows the confirmation of *E. histolytica* and the differentiation from *E. dispar* in human feces [8],[9]. Based on this method, a unique cohort of asymptomatically infected *E. histolytica* carriers was identified during an extensive epidemiologic study performed in a region of high incidence of amebiasis in central Vietnam and further investigated in the present study [6],[9].

Of particular interest is whether these asymptomatically infected individuals develop antibody-mediated immune responses against *E. histolytica*, and whether these responses differ from those of subjects with ALA. Invasive amebiasis is associated with the development of high anti-amebic immunoglobulin G (IgG) titers. For diagnostic purposes, the usage of a soluble amebic protein (SAP) preparation reveals high sensitivity and specificity [10]. Humoral immune responses against non-proteinaceous molecules of *E. histolytica* like the lipophosphoglycans, that differ in their composition from apathogenic amebaes, might also represent interesting antigens for the investigation of serum responses in AC and ALA patients [11],[12]. Despite the development of high and long lasting specific antibody titers during invasive amebiasis, reinfections frequently occur [3]. Therefore, a more detailed analysis of the humoral immune response analyzing the IgG subclasses might give additional information associated with resistance or susceptibility to invasive disease.

The four different IgG subclasses represent different immune mechanisms for combating infection: IgG1 and IgG3 recognize microbial proteins, while IgG2 preferentially binds microbial carbohydrate antigens [13],[14]. IgG4 only weakly responds to many antigens and possesses a blocking activity to IgG1- and IgE-mediated immune functions [15]. In contrast to IgG2 and IgG4, IgG1 and IgG3 drive inflammatory processes and antigen clearance through their high affinity to C1q [16] and binding to FcyRI, FcyRII, and FcyRIII expressed on neutrophils, natural killer cells, and tissue macrophages [17]. IgG1 shows the longest half-life of all IgG subclasses [18], as well as strong complement-binding activity. Therefore, IgG1 could represent a major arm of the host defense against complement-sensitive *E. histolytica* trophozoites [19],[20].

In addition to the humoral immune response, serum levels of mediators of cellular immunity may also be suitable for elucidating immune mechanisms underlying the outcome of an infection. Apart from the pro- and anti-inflammatory cytokines, chemokines involved in innate immune mechanisms, especially the C-C chemokine ligands (CCL)2 and CCL3, are of interest. CCL2, formerly known as monocyte chemotactic protein (MCP-1), is critically involved in the immunopathology mediated by inflammatory monocytes in many human diseases and murine models for human diseases [21], including the mouse model of ALA [22]. CCL3, formerly known as macrophage inflammatory protein (MIP)-1α, and CCL4 (MIP-1β) are produced by a variety of immune cells, including macrophages and lymphocytes, and are involved in the recruitment and activation of polymorphonuclear leukocytes [23]. Moreover, it has recently been shown that estradiol treatment down-regulates CCL2 and up-regulates CCL3 production, thereby reducing the severity of influenza infection in female mice [24].

In the present study, we examined serum immune markers associated with resistance and susceptibility in two unique cohorts of individuals infected with *E. histolytica*: asymptomatic carriers of *E. histolytica* and ALA patients. In addition, we also analyzed the results with respect to the sex of the individuals.

## Methods

### Study population

The sera investigated in the present study were initially collected in the context of two previous studies, a clinical investigation focused on ALA treatment, and a longitudinal study of intestinal *E. histolytica* infections in an area in central Vietnam that is endemic for amebiasis [3],[25]. Sera were obtained from patients upon admittance and diagnosis of ALA at the Central Hospital of Hué, Vietnam (n = 38; 8 women, 30 men; age: 31–58 years; mean: 43); from asymptomatic, healthy *E. histolytica* carriers (n = 44; 28 women, 16 men; age: 21–60; mean = 37.6); and from healthy individuals infected with *E. dispar* (n = 24; 19 women, 5 men; age: 22–60; mean = 42.1). The diagnosis of ALA was based on clinical signs, ultrasound findings, sterility of abscess pus, and a good response to therapy with metronidazole, i.e., the disappearance of clinical symptoms and a reduction in abscess size. The samples from ALA patients were taken at the time point of diagnosis. Intestinal infection with *E. histolytica* and *E. dispar* in asymptomatic carriers was diagnosed using species-specific PCR and DNA extracted directly from fecal samples [3],[9]. Individuals with a fecal PCR result positive for *E. dispar* has been shown previously to reveal the same percentage of positive serology to *E. histolytica* as individuals negative to *E. histolytica* and *E. dispar* [9].

The study was approved by the Scientific Council of Education, Training, and Ethics of Hué Medical School, Vietnam, on September 11, 1998, a written informed consent for participation was obtained from participants.

### Antigens used for ELISA

Soluble amebic proteins (SAP) were prepared from the supernatant of axenically-grown *E. histolytica* trophozoites (HM-1:IMSS) following several steps of freezing and thawing [26] and centrifugation at 300 × g. Protein concentrations were determined using a bicinchoninic acid (BCA) protein assay kit (Pierce, Rockford, IL, USA). *E. histolytica* lipopeptidophosphoglycan (*Eh*LPPG) was purified from the membranes of *E. histolytica* trophozoites [27]. In brief, trophozoites of the late logarithmic phase of growth were washed, resuspended in pyrogen-free water, and lysed by freezing and thawing. The homogenate was centrifuged at 430 × *g* at 4°C for 10 min. Subsequently, the supernatant was recovered and the trophozoite membranes were enriched by ultracentrifugation at 150,000 × *g* for 40 min. The obtained pellet was extracted with a mixture of chloroform/methanol/water 10:10:3 (by volume) and the insoluble material was recovered by centrifugation, dried, resuspended in distilled pyrogen-free water, and extracted three times with an equal volume of 90% phenol at 68°C for 30 min with constant stirring. The aqueous phase containing *Eh*LPPG was recovered after centrifugation at 12,000 × *g* for 30 min and dialysis against distilled water. The *Eh*LPPG was dried, weighed, and diluted to a concentration of 1 mg/ml in water.

### Measurement of ameba-specific total immunoglobulin (IgGt) and IgG subclasses (IgG1, IgG2, IgG3, and IgG4), and IgA

Semi-quantitative analysis of antibody titers to soluble amebic proteins was performed using ELISA as previously described [10] with modifications as described in the following. Serum dilutions were previously determined to be optimal according to the different amounts for each subclass within the serum. Briefly, SAP at a concentration of 2 μg/ml and *Eh*LPPG (0.0625 μg/ml) were used to coat flat-bottomed microtiter plates (Nunc, Roskilde, Denmark) overnight at 4°C in carbonate buffer at pH 9.6. The plates were washed three times with PBS containing 0.05% Tween 20, nonspecific binding sites were blocked by 5% bovine serum albumin in PBS for 90 min, and the plates were washed as before.

For the SAP ELISA, patients' sera were added at the following dilutions in 0.05% BSA/PBS: 1:800, 1:3,200, and 1:12,800 for IgG_t_; 1:600, 1:1,800, and 1:5,400 for IgG1 and IgG4; 1:200, 1:600, and 1:1,800 for IgG2 and IgG3; and 1:100, 1:400, and 1:1,600 for IgA. The prepared plates with the diluted sera were then incubated overnight at 4°C. For the *Eh*LPPG-ELISA, the following dilutions were used for the measurement of IgG_t_ and IgG2. For IgG_t_, sera were diluted as for the measurement of IgG_t_ in the SAP ELISA (1:800, 1:3,200; 1:12,800); for IgG2, sera were diluted at 1:50, 1:100, 1:400 and 1:1,600. After washing, class- and subclass-specific mouse monoclonal anti-human immunoglobulin antibodies (HP6070A for IgG1 and HP6025 for IgG4, both diluted 1:2,000; HP6002 for IgG2 and HP6050 for IgG3, both diluted 1:1,000; clone 6E2C1 for IgA, diluted 1:500 in 0.05% BSA/PBS) were incubated for 90 min at room temperature. The IgG subclass antibodies were obtained from Calbiochem (La Jolla, CA, USA) while the total IgG antibodies were obtained from DAKO (Glostrup, Denmark). Plates were washed, then alkaline phosphate-conjugated goat anti-mouse IgG (Dianova, Hamburg, Germany) was added at a dilution of 1:5,000 in 0.05% BSA/PBS and the reaction was incubated for 45 min at room temperature. The plates were again washed and developed using *p*-nitrophenyl phosphate (Sigma, Deisenhofen, Germany). Endpoint titers were estimated by extrapolation of the OD values obtained for the different serum dilutions and for each antibody isotype using the bivariate scattergram of StatView.

### Serum analytes

Serum samples were tested for 14 analytes (granulocyte macrophage colony-stimulating factor (GM-CSF), IL-1β, IL-4, IL-6, IL-10, IL-12p70, IL-13, IL-17, IL-23, interferon (IFN)-γ, CCL2, CCL3, CCL4, and tumor necrosis factor (TNF)-α using custom testing services based on a flow cytometry bead-based multiplex immunoassay panel (provided by BioLegend, Inc., San Diego, CA). Briefly, antibodies specific for the 14 analytes were conjugated to 14 different fluorescence-encoded beads. The beads were mixed with serum samples (diluted 2-fold), incubated with shaking for 2 hours at room temperature, washed, and incubated with a cocktail of 14 different biotinylated detection antibodies for 1 hour. Finally, streptavidin-PE was added for 30 min and the beads were washed and analyzed. Due to restricted sample volumes, analysis was only possible for 34 sera from ALA patients (7 women, 27 men), 41 sera from AC patients (25 women, 16 men) and 24 sera from *E. dispar* carrier (19 women, 5 men).

### Statistical analysis

The ALA, AC, and *E. dispar-*infected groups were compared in terms of antibody titers and Th1/Th2 cytokine and chemokine levels by using the Kruskal-Wallis test followed by the Mann–Whitney U test with Bonferroni correction. To evaluate differences between male and female ALA and AC subjects in terms of IgG titers, the Mann–Whitney U test was used. To evaluate differences between female and male ALA, AC, and *E. dispar* subjects in terms of pro- and anti-inflammatory cytokine and chemokine levels, the Mann–Whitney U test with Bonferroni correction was performed. Differences were considered to be significant if p-values were: *p < 0.05, **p < 0.005, and ***p < 0.0005.

## Results

### Antibody class and subclass responses to soluble amebic proteins (SAP) were higher in individuals infected with *E. histolytica* than in those infected with *E. dispar*

**Figure 1:**
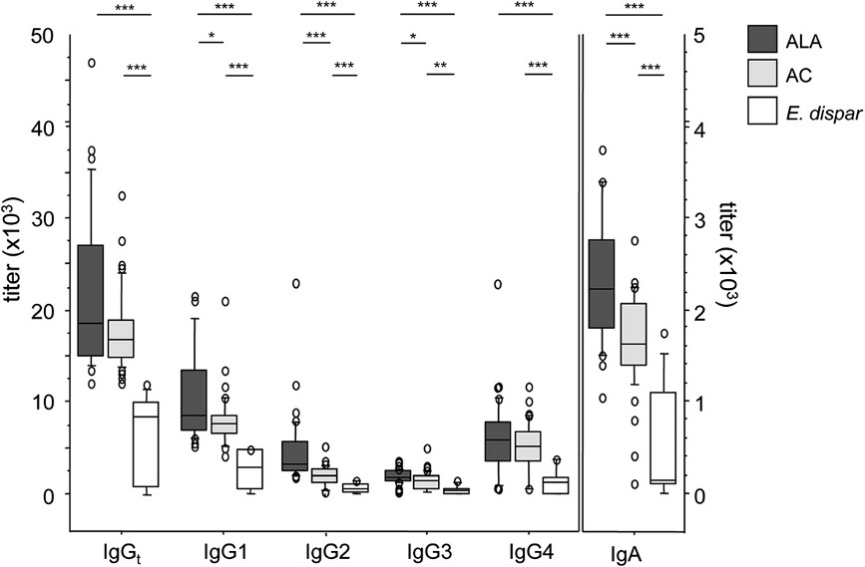
**Serum antibody titers against soluble amebic proteins (SAP) in individuals infected with*E. histolytica*or*E. dispar*.** Anti-amebic total immunoglobulin G (IgG_t_), IgG1, IgG2, IgG3, and IgG4 subclasses, and IgA titers were measured in 38 patients with amebic liver abscess (ALA), 44 asymptomatic *E. histolytica* carriers (AC), and 24 individuals infected with the apathogenic *E. dispar*. Statistics: Kruskal-Wallis test followed by Mann–Whitney *U* test with Bonferroni correction (*p < 0.05, **p < 0.005, and ***p < 0.0001).

The levels of total IgG (IgG_t_), subclasses IgG1, IgG2, IgG3, and IgG4, and IgA that were reactive with SAP were analyzed in the sera of 82 individuals with a proven *E. histolytica* infection and 24 subjects infected with *E. dispar*, both groups verified by stool PCR. The levels of immunoglobulins were calculated as ELISA titers. IgA was included in the analysis since IgA represents the antibody class associated with exposed mucosa surfaces. Significantly higher reactivities of all examined antibody types and isotypes were found in *E. histolytica*-infected individuals - in both ALA patients and AC - than in individuals infected with *E. dispar* (p < 0.0003; p < 0.0036) (Figure 1). ALA patients and AC did not differ in their *E. histolytica*-specific IgG_t_ (p = 0.094) and IgG4 (p = 0.2) titers, but did differ in their IgG1 (p < 0.034) and IgG3 titers (p < 0.033) with higher titers obtained for ALA patients. The strongest differences were found between the IgG2 titers of ALA patients and AC (p < 0.0003).

In addition to SAP, we introduced a non-proteinaceous, major amebic antigen preparation consisting of membrane bound glycolipids, the *Eh*LPPGs, to analyze the humoral immune responses in the three study groups.

**Figure 2:**
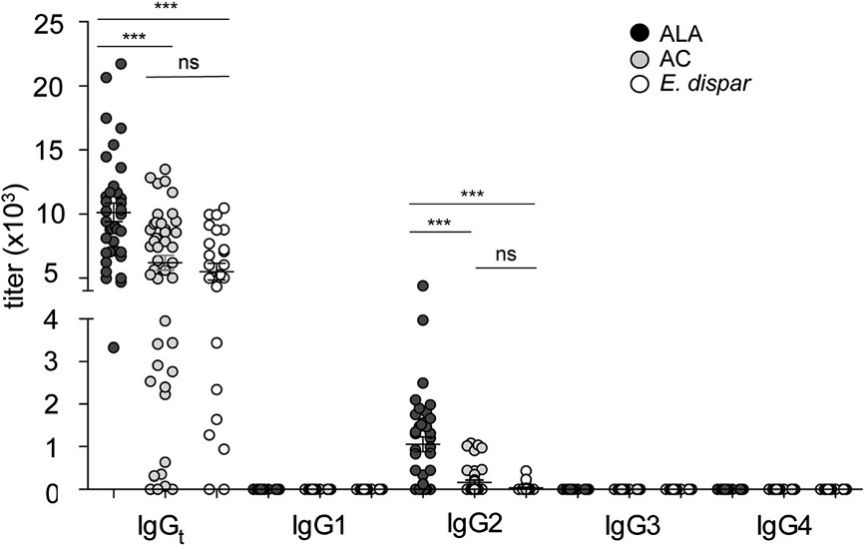
***Eh*LPPG-specific total immunoglobulin G (IgG**_**t**_**), IgG1, IgG2, IgG3, and IgG4 antibody titers in subjects infected with*E. histolytica*or*E. dispar*.** The sera were from 38 patients with amebic liver abscess (ALA), 44 asymptomatic *E. histolytica* carriers (AC), and 24 individuals infected with the apathogenic *E. dispar*. The IgG_t_, IgG1, IgG2, IgG3, and IgG4 titers of the three study groups are shown. Statistics: Kruskal-Wallis test followed by Mann–Whitney *U* test, ***p < 0.0001; ns, not significant. ALA, amebic liver abscess; AC, asymptomatic *E. histolytica* carrier; *E. dispar*, individuals infected with *E. dispar*.

The investigation revealed that the serum titers to *Eh*LPPG were higher in ALA patients compared with both AC (p < 0.0003) and *E. dispar* controls (p < 0.0003), while no differences in titers against *Eh*LPPG were found between the AC and the *E. dispar*-infected individuals (p = 0.27) (Figure 2). Of the four IgG subclasses, only IgG2 subclass antibodies were positive, and they showed the same group distribution as found for IgGt. IgG2 titers were highest in ALA patients; significantly higher than in AC (p < 0.0003) and *E. dispar* controls (p < 0.0003), while the serum titers from AC and *E. dispar*-infected individuals did not differ from each other (p = 0.17).

### Liver and asymptomatic infections with *E. histolytica*are associated with immune responses skewed towards an anti-inflammatory immune response

**Figure 3:**
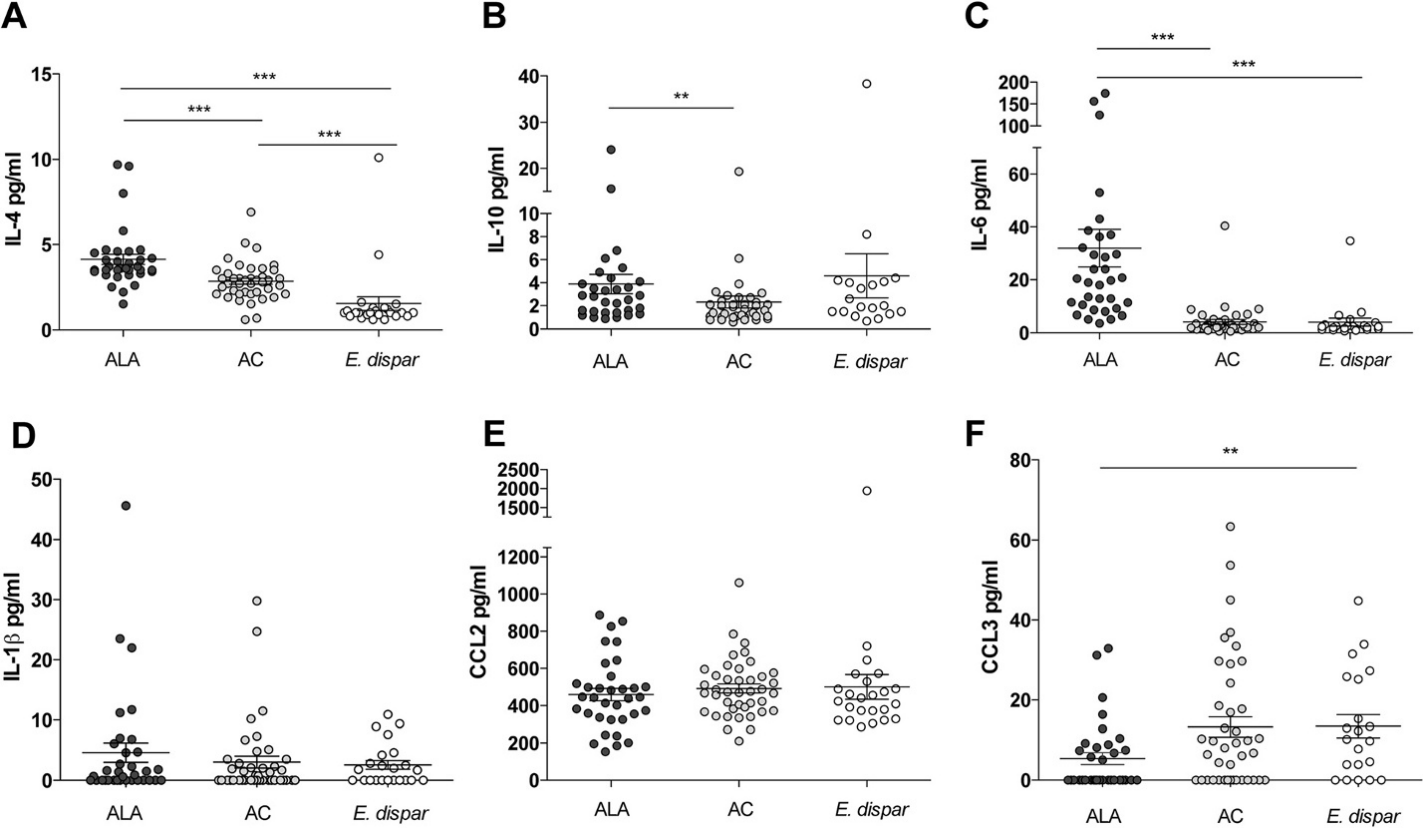
**Serum cytokine and chemokine levels in individuals infected with*E. histolytica*or*E. dispar*.** The sera were from 34 patients with amebic liver abscess (ALA), 41 asymptomatic *E. histolytica* carriers (AC), and 24 individuals infected with the apathogenic *E. dispar*. Cytometric bead array analyses were used to determine the serum concentrations of IL-4, IL-10, IL-6, IL-1β, chemokine (C-C motif) ligand 2 (CCL2), and CCL3 in the sera from ALA patients, AC, and *E. dispar*-infected subjects **(A–F)**. Statistics: Kruskal-Wallis test followed by Mann–Whitney *U* test with Bonferroni's correction (*p < 0.05, **p < 0.005, and ***p < 0.0001); ns, not significant. ALA, amebic liver abscess; AC, asymptomatic *E. histolytica* carrier; *E. dispar*, individuals infected with *E. dispar*.

We measured IL-1β, IL-4, IL-6, IL-10, IL-12p70, IL-13, IL-17, IL-23, IFN-γ, and TNF-α, as well as CCL2, CCL3, and CCL4 in patient sera using a cytometric bead array. Of all the cytokines tested, only IL-4, IL-10, and IL-6 serum levels differed among the three study groups, and were significantly higher in ALA patients than in AC (IL-4: p < 0.0003; IL-10: p < 0.0017; IL-6: p < 0.0003) and *E. dispar*-infected individuals (IL-4: p < 0.0003; IL-10: p = 0.21; IL-6: p < 0.0003) (Figure 3A–C). CCL2 serum levels did not differ among the three study groups, but CCL3 was significantly lower in ALA patients than in *E. dispar*-infected individuals (p < 0.02). CCL3 levels in ALA patients were also lower than those in AC, but this difference was not quite statistically significant (p = 0.057 (Figure 3E–F)).

The cytokines IL-1β (Figure 3D), IL-12p70, IL-13, IL-17, IL-23, and TNFα did not differ between the study groups. IFNγ and GM-CSF were under the detection limit (data not shown).

### Sex-related differences in sera from ALA patients, AC, and *E. dispar*-infected individuals

**Figure 4:**
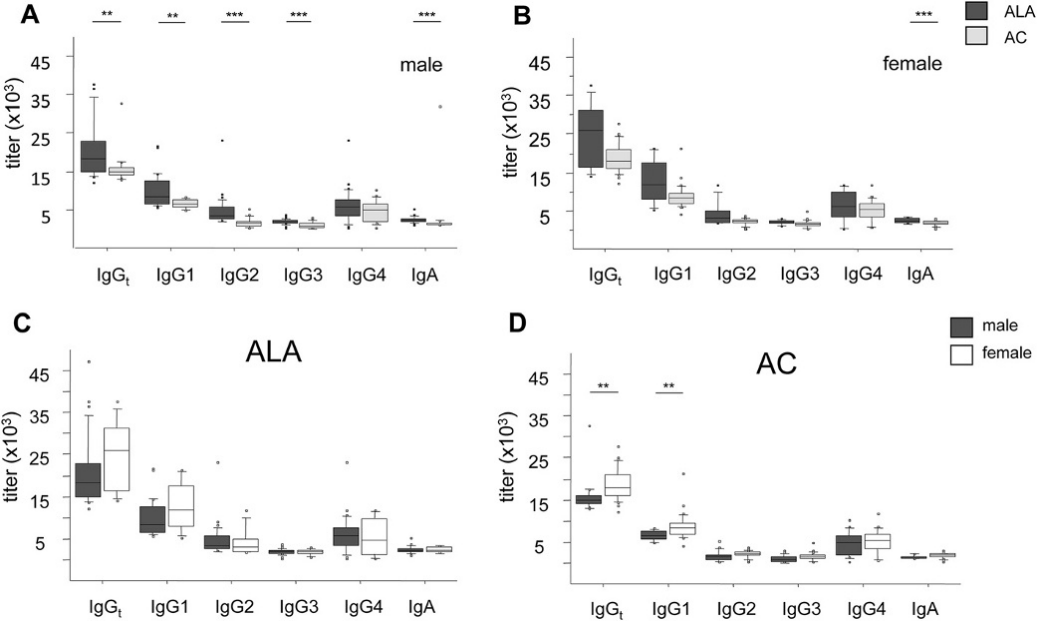
**Sex-related analysis of serum antibody titers against soluble*E. histolytica*amebic proteins (SAP) in individuals infected with*E. histolytica*.** Differences in titers of anti-amebic total immunoglobulin IgG (IgG_t_), IgG subclasses, and IgA between 38 ALA patients and 44 asymptomatic *E. histolytica* carriers (AC) were compared in males **(A)**, and females **(B)**. Differences in anti-amebic total immunoglobulin G (IgG_t_), IgG subclasses, and IgA titers between males and females were compared in ALA patients **(C)** and AC **(D)**. Statistics: Mann–Whitney *U* test (*p < 0.05, **p < 0.005, and ***p < 0.0001). ALA: male n = 30, female n = 8; AC: male n = 16, female n = 28.

Analysis of total serum IgG and IgG subclasses against SAP within the same sex revealed that male ALA patients had significantly higher IgG_t_ (p < 0.009), IgG1 (p < 0.022), IgG2 (p < 0.0001), IgG3 (p < 0.0001), and IgA (p < 0.0001) compared with male AC. However, the IgG4 titers for these two groups did not differ (Figure 4A). By contrast, there were no significant differences in the titers of IgGt and IgG subclasses against SAP between female ALA patients and female AC, except for IgA, which showed higher titers in female ALA patients than in female ACs (p < 0.0001) (Figure 4B).

Comparison of IgG_t_ and IgG subclass titers between women and men in the ALA and AC groups revealed that, among the ALA patients, the antibody responses did not differ significantly between male and female patients (Figure 4C). In the AC group, significantly higher IgG_t_ and IgG1 antibody titers were found in women compared with men (p *<* 0.002; p < 0.002). IgG2, IgG3, and IgG4 titers, as well as IgA titers, did not differ between the sexes in the AC group (Figure 4D). Distributing the IgG titers against *Eh*LPPG by sex, we found no differences in the IgG_t_ titers between male and female ALA patients and AC, and only a trend towards higher IgG2 titers to *Eh*LPPG in male ALA patients (p = 0.072) and male AC (p = 0.058) compared with their female counterparts (data not shown).

**Figure 5:**
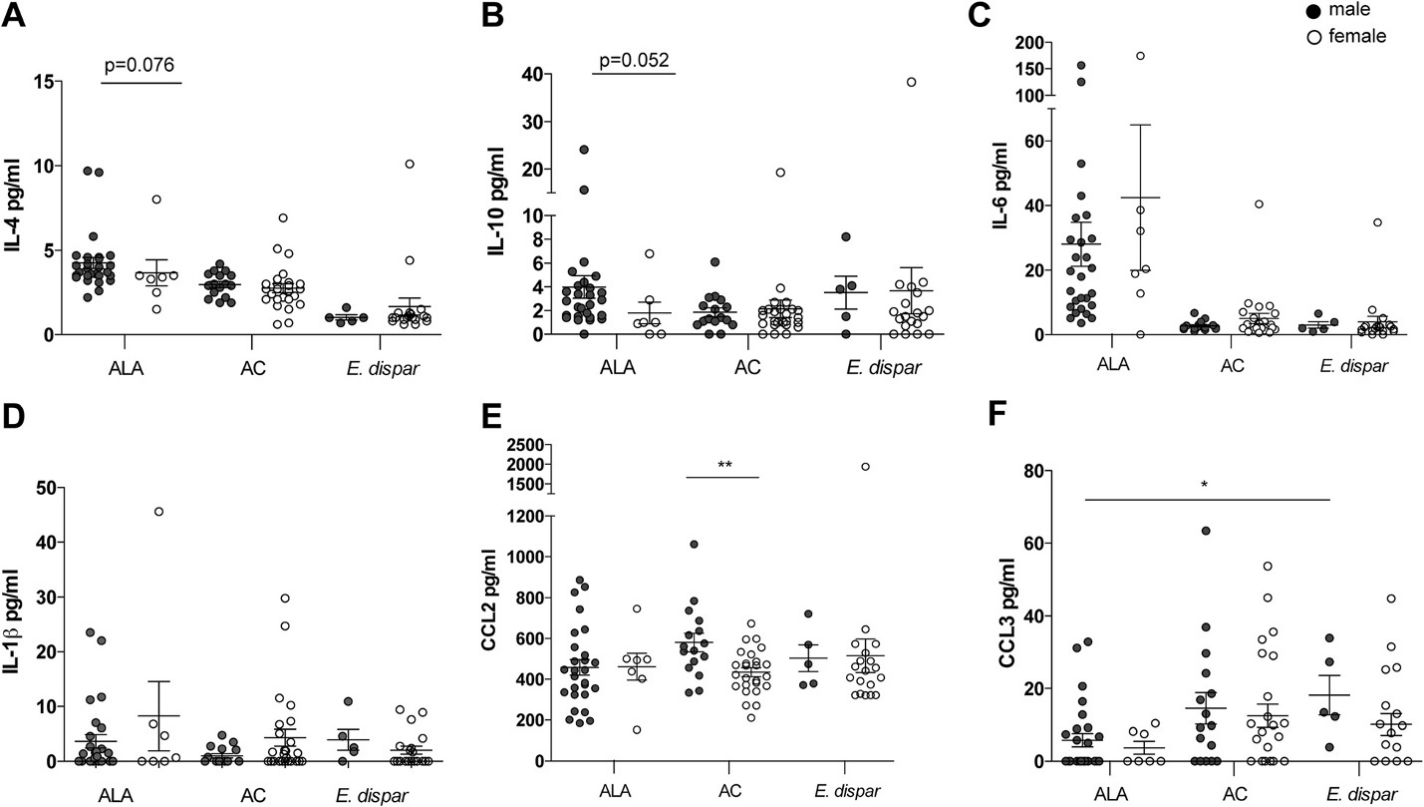
**Sex-related analysis of serum cytokine and chemokine levels in individuals infected with*E. histolytica*or*E. dispar*.** The sera were from 34 patients with amebic liver abscess (ALA), 41 asymptomatic *E. histolytica* carriers (AC), and 24 individuals infected with the apathogenic *E. dispar*. Differences between males and females in the serum levels of IL-4, IL-10, IL-6, IL-1β, chemokine (C-C motif) ligand 2 (CCL2), and CCL3 in ALA patients, AC and *E. dispar*-infected subjects are shown **(A–F)**. ALA: male n = 27, female n = 7; AC: male n = 16, female n = 25; *E. dispar*: male n = 5, female n = 19. Statistics: Mann–Whitney *U* test with Bonferroni correction was used when comparing more than two groups; Mann–Whitney *U* test was used when comparing only two groups (*p < 0.05 and **p < 0.005).

Analyzing the serum cytokines IL-4, IL-10, IL-6, and IL-1β with regard to sex within the ALA, AC, and *E. dispar* groups, we only found a trend towards higher IL-4 and IL-10 in male ALA patients compared with female ALA patients (p = 0.076; p = 0.052) (Figure 5A–B). No sex-related differences were seen for IL-6 and IL-1β (Figure 5D–E). Most interestingly, a significant sex-related difference was found in CCL2, with higher serum levels in male AC than in female AC (p < 0.0033) (Figure 5E). We found no sex-related differences in the serum levels of CCL3, though male ALA patients exhibited lower levels of CCL3 than male *E. dispar*-infected individuals (p < 0.02) (Figure 5F). CCL4 was detectable in all study groups, with no differences between the sexes (data not shown).

## Discussion

The present study was performed to identify immunological variables in two *E. histolytica-*infected study populations from the same endemic region of Vietnam. The population of this region included ALA patients [25] and people infected with *E. histolytica* who did not show any signs of disease. Residents from the same region who were infected with the nonpathogenic ameba species *E. dispar* served as a control group [3]. A comparison of immunological parameters among these groups may provide important information needed to address why certain individuals are able to control an intestinal *E. histolytica* infection while others are not, and why men, despite similar infection rates in women, are more susceptible to the development of amebic liver abscesses [4]-[6]. The immunological variables studied included *E. histolytica*-specific IgG and the various IgG subclasses, along with IgA and serum cytokine and chemokine levels.

Our analysis of the humoral immune responses to *E. histolytica* showed that, of the three groups, the patients with ALA had the highest anti-*E. histolytica* IgG antibody titers. It has been previously observed that ALA patients, like patients with amebic colitis, have high anti-*E. histolytica* IgG antibody titers [28],[29], but this is the first time that such patients have been compared with clearly defined AC subjects. Another finding in this study was that the AC subjects developed high anti-*E. histolytica* IgG_t_ titers and high titers of the IgG subclasses IgG1, IgG2, IgG3, and IgG4, as well as IgA, which significantly differed from individuals infected with the apathogenic *E. dispar*. As previously shown, the *E. dispar* positive individuals employed in the present study exhibited antibody positivity to *E. histolytica* to the same extend as *E. histolytica* or *E. dispar* negative individuals (*E. dispar* PCR positive: 20.8%; *E. histolytica - E. dispar* PCR negative: 18.3% vs. *E. histolytica* PCR positive: 82.6%) [9]. From this study we conclude that the observed titers in *E. dispar* positive individuals represent the background titers against *E. histolytica* found in an amebiasis endemic area.

The presence of an *E. histolytica-*specific antibody response in AC has been reported earlier. However, in one study, PCR confirmation of the parasite was not available at that time and the study group was comparatively small [30]. From a serologic investigation of *E. histolytica* cyst passers that were identified during an *E. histolytica* outbreak in an institution for mentally retarded individuals in Japan, a weak but positive serological result was detected in 82% (27/33) of the asymptomatically infected patients. Details on the serologic results in this study were not communicated, and further subclass analysis was not performed [31]. Taken together, the presence of an *E. histolytica-*specific humoral immune response suggests that AC subjects might be continuously antigenically stimulated by their *E. histolytica* infection, either due to the induction of subclinical mucosal lesions or by the occasional, but controlled, spread of *E. histolytica* trophozoites into the liver.

Another finding of our study was that female AC subjects had significantly higher anti-*E. histolytica* IgG_t_ and IgG1 levels than male AC subjects. This tendency was also found in female ALA patients, however, due to the low number of ALA cases in women, this result revealed no significance.

IgG1 is known to be a strong complement activator, since it binds to C1q, and since the complement system is a major part of the host innate immune defense mechanism against *E. histolytica* trophozoites, which are highly sensitive to complement-mediated lysis [32]-[34]. In addition, a more recent study showed that serum from women kills *E. histolytica* trophozoites significantly more effectively than serum from men, and that the mechanisms involve complement-mediated amebic lysis [35]. Another IgG1-related mechanism that may promote *E. histolytica* trophozoite killing is that monocytes, macrophages, neutrophils, and dendritic cells bear high-affinity receptors for IgG1 (and IgG3). These cells are stimulated in the presence of IgG1/IgG3 to express *i*NOS and therefore generate host cell cytotoxicity against amebic trophozoites [36],[37]. Taken together, this would suggest that although subclinical invasion occurs continuously in women, it is controlled by their strong humoral IgG immune response, thereby suppressing the development of ALA at an early stage. Once ALA occurs, we assume that ALA pathology proceeds equally in men and women, despite high antibody titers in women suggesting that the immune response during the asymptomatic *E. histolytica* infection decides the outcome of the disease.

We also investigated the responses to *Eh*LPPG glycolipid, which is abundant on the surface of *E. histolytica* trophozoites and consists of carbohydrates and lipids [11],[27],[38]. The study subjects mounted a different response to this antigen compared with their response to the whole protein extract SAP. Whereas the AC group responded almost as well as the ALA group to SAP and *Eh*LPPG, the *E. dispar* group response was lower. By contrast, patients with ALA had higher IgG_t_ titers against *Eh*LPPG than either the AC or the *E. dispar* group. The AC and *E. dispar* groups had similar, low *Eh*LPPG-specific IgG_t_ levels. From all IgG subclasses, only IgG2 was reactive with *Eh*LPPG, which is consistent with the fact that IgG2 specifically recognizes carbohydrates. The higher antibody response against *Eh*LPPG in ALA patients could be indicative of a more intense engagement of the immune system with the pathogen in the liver during invasive disease.

The serum cytokine and chemokine levels in the ALA, AC, and *E. dispar* groups were also analyzed in the present study. Interestingly, the Th2-type cytokine IL-4 was strongly associated with *E. histolytica* infection. ALA patients as well as AC subjects had significantly higher IL-4 levels than the *E. dispar*-infected individuals, suggesting that asymptomatic infection with *E. histolytica* induces a considerable immune response. A recent comparison of a smaller number of ALA patients, ACs, and *E. dispar-*infected individuals showed a comparable trend for IL-4. IL-4 mRNA expression of peripheral blood lymphocytes (PBMC) stimulated with amebic extract was observed in 3 of 5 subjects with ALA and 2 of 5 AC subjects, but also in 1 of 5 *E. dispar* carriers. This study also showed that all ALA subjects, but none of the AC or *E. dispar*-infected subjects, were positive for IL-10 mRNA expression by stimulated PBMCs [39]. The latter result was confirmed by our study, which found that IL-10 serum levels were significantly increased only in ALA patients and not in the other groups, further indicating that invasion of the liver tissue by *E. histolytica* elicits a anti-inflammatory immune response. As in the study of Bansal et al., pro-inflammatory cytokines like IFNγ were poorly detectable during ALA or asymptomatic *E. histolytica* infection.

The cytokine IL-6 was found at significantly higher levels in ALA patients compared with AC or *E. dispar*-infected individuals. IL-6 is involved in the production of the acute phase reactant C-reactive protein (CRP) in the liver via IL-1β and STAT3 [40]. Further, both IL-6 and CRP represent peripheral markers for acute inflammation in many human disorders [41],[42]. Consistently, sera from the same ALA patients as used in the present study showed elevated CRP values (210–242 mg/ml) that decreased upon anti-amebic treatment [25]. Most recently, it was shown that *E. histolytica* possesses a human-like macrophage migration inhibitory factor (EhMIF) that induces the production of IL-6 that might further contribute to IL-6 production during invasive amebiasis [43]. In a mouse model of amebiasis, IL-6 deficiency increased the susceptibility to ALA, suggesting a relevant role for IL-6 in the inflammatory processes induced by *E. histolytica* [44].

Our analysis of the serum chemokine levels in the ALA, AC, and *E. dispar* groups revealed several interesting aspects. First, the three groups had equivalently high levels of CCL2, but high serum levels of CCL2 had also been shown in other healthy control groups [45],[46]. Second, sex analyses revealed that the male AC subjects had significantly higher CCL2 levels than female AC. A study with a murine model of ALA showed that CCL2 participates in the immunopathology of ALA through recruitment of inflammatory, tissue-destructive monocytes [22]. Because men have higher monocyte counts than women [47], an additional increase in the monocyte chemotactic CCL2 may indicate that men are more likely than women to recruit inflammatory tissue-destructive monocytes before *E. histolytica* spreads into the liver. Furthermore, the percentage of circulating monocytes of peripheral blood mononuclear cells in men is higher than in women and monocytes in males exhibit a stronger innate immune response than those in females [48],[49]. However, once *E. histolytica* has initiated the liver damage, CCL2 is equally responsible for monocyte recruitment in both men and women.

ALA patients showed significantly lower CCL3 levels compared with *E. dispar* subjects and a strong tendency (p = 0.057) towards a lower CCL3 expression level compared with AC, which suggests that activated neutrophils may play a role in the control of *E. histolytica* gut infection. This notion is supported by *in vitro* and *in vivo* studies that show that activated human or murine neutrophils exhibit amebicidal activity [50],[51].

In summary, the present study supports the notion that the strong antibody responses found in AC reflect intense interactions between the host immune system and the invading pathogen in the absence of clinical symptoms. In addition, sex-related analyses revealed that female AC subjects expressed higher levels of anti-*E. histolytica* IgG1 than male AC, which suggests that resistance to ALA may be associated with complement-mediated mechanisms. The predisposition of males towards immunopathology via increased CCL2 levels during intestinal *E. histolytica* infection may reflect their greater tendency to recruit inflammatory monocytes.

## Conclusion

Astonishing little is known about the immune response underlying invasive amebiasis. This includes all stages of infection, ranging from asymptomatic colonization up to the invasive stages like amebic colitis or amebic liver abscess. In addition, the pronounced sex bias in the outcome of amebic liver abscess is nearly uninvestigated although sex dependent differences in susceptibility and resistance to many infections become unquestionably an increasing field of interest [52],[53]. Nowadays, new methods accurately allow the diagnosis of *E. histolytica* infections, thus overcoming deficiencies of many epidemiological studies in the past and enables and justifies state-of-the-art immunological investigations. Performing respective immunological studies on a disease like amebiasis, that offers such clearly distinguishable patient groups, could give interesting clues about the outcome of the disease amebiasis, but also on mechanisms determining sex-specific immunity in a wide spectrum of diseases.

## Authors’ original submitted files for images

Below are the links to the authors’ original submitted files for images. Authors’ original file for figure 1 Authors’ original file for figure 2 Authors’ original file for figure 3 Authors’ original file for figure 4 Authors’ original file for figure 5
