# Supplementary figures and images for: Immune markers characteristic for asymptomatically infected and diseased Entamoeba histolytica individuals and their relation to sex
Source: BMC Infect Dis. 2014 Nov 25;14:621. doi: 10.1186/s12879-014-0621-1 (PMC4252988; doi:10.1186/s12879-014-0621-1)

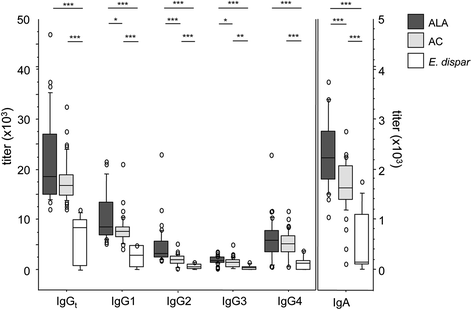

Supplement: Supplementary file 1 — Authors’ original file for figure 1 [file 12879_2014_621_MOESM1_ESM.gif]

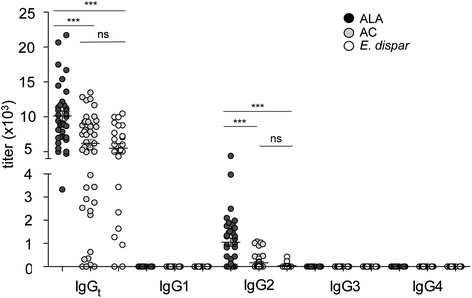

Supplement: Supplementary file 2 — Authors’ original file for figure 2 [file 12879_2014_621_MOESM2_ESM.gif]

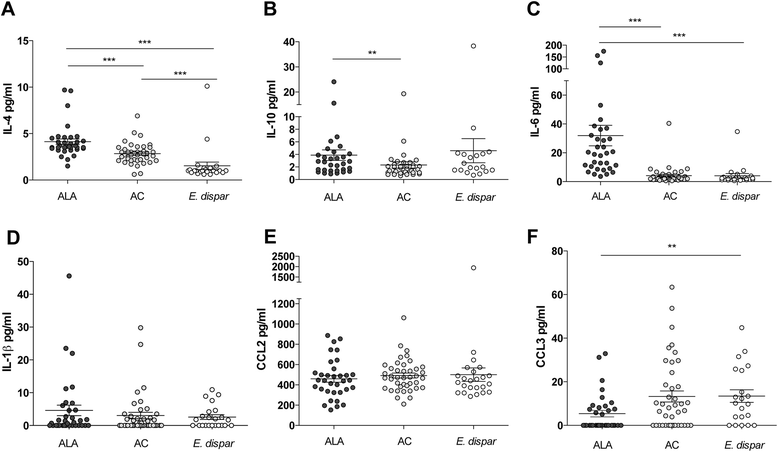

Supplement: Supplementary file 3 — Authors’ original file for figure 3 [file 12879_2014_621_MOESM3_ESM.gif]

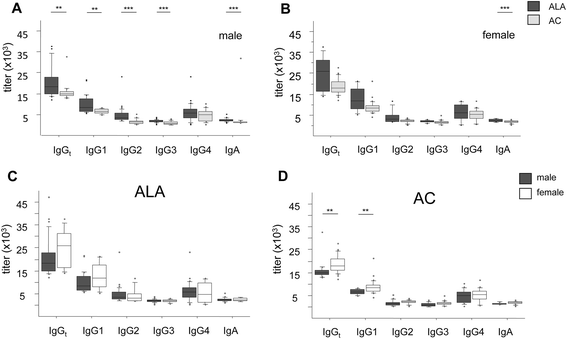

Supplement: Supplementary file 4 — Authors’ original file for figure 4 [file 12879_2014_621_MOESM4_ESM.gif]

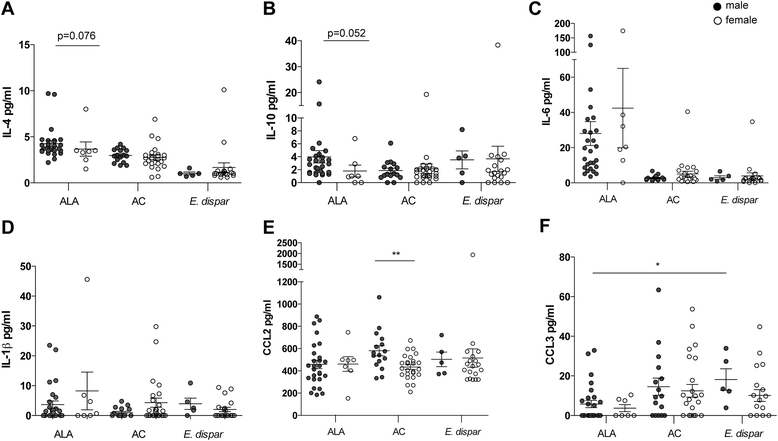

Supplement: Supplementary file 5 — Authors’ original file for figure 5 [file 12879_2014_621_MOESM5_ESM.gif]
